# Safe percutaneous suprapubic catheterisation

**DOI:** 10.1308/003588412X13373405385412

**Published:** 2012-05

**Authors:** NK Goyal, A Goel, SN Sankhwar

**Affiliations:** CSM Medical University,India

**Keywords:** Catheter, Complications, Cystostomy, Suprapubic cystostomy

## Abstract

**INTRODUCTION:**

We describe our technique of percutaneous suprapubic catheter insertion with special reference to steps that help to avoid common complications of haematuria and catheter misplacement.

**METHODS:**

The procedure is performed using a stainless steel reusable trocar under local infiltrative anaesthesia, usually at the bedside. After clinical confirmation of a full bladder, the trocar is advanced into the bladder through a skin incision. Once the bladder is entered, the obturator is removed and the assistant inserts a Foley catheter followed by rapid balloon inflation. Slight traction is applied to the catheter for about five minutes. Patients with previous lower abdominal surgery, an inadequately distended bladder or acute pelvic trauma do not undergo suprapubic catheterisation using this method.

**RESULTS:**

The procedure was performed in 72 men (mean age: 42.4 years, range: 18–78 years) with urinary retention with a palpable bladder. The average duration of the procedure was less than five minutes. No complications were noted in any of the patients.

**CONCLUSIONS:**

Trocar suprapubic catheter insertion is a safe and effective bedside procedure for emergency bladder drainage and can be performed by resident surgeons. The common complications associated with the procedure can be avoided with a few careful steps.

Percutaneous suprapubic catheterisation (SPC) is a frequently performed and well established procedure for urinary drainage. Despite this, there is a paucity of data regarding the procedure. This was highlighted in the guidelines published by the British Association of Urological Surgeons (BAUS).^1^ Generally, it is believed that SPC is associated with minimal complications and morbidity. However, complications do occur and include bleeding, clot retention and catheter dislodgement as early complications,^2,3^ prompting BAUS to recommend that it should be performed only by surgeons with appropriate skills.^1^ Nevertheless, these complications are avoidable if the procedure is performed carefully. With this in mind, we describe the technique of bedside, percutaneous SPC with reusable instruments, emphasising how to avoid these complications.

## Technique

**Figure 1:**
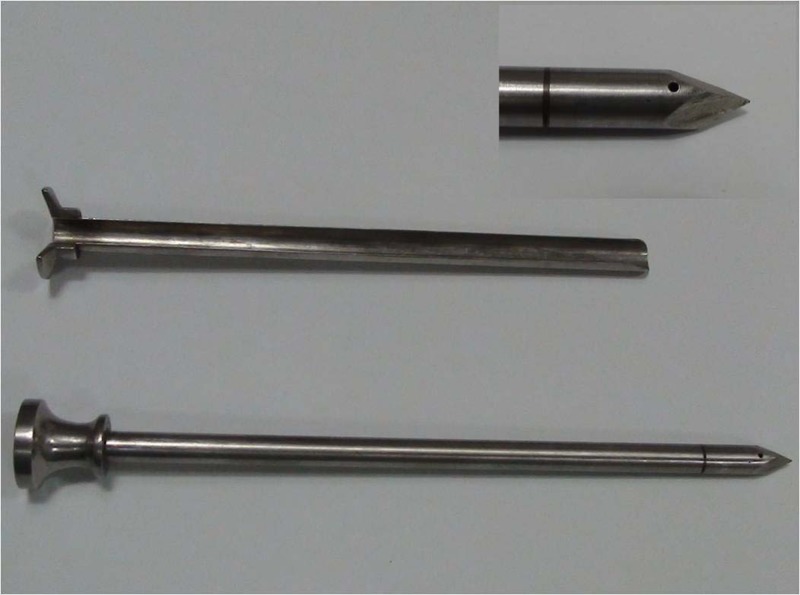
Commercially available stainless steel trocar with an outer sheath and inner obturator. Inset shows distal end of trocar with a hole near the tip.

After explaining the procedure to the patient and relatives, written consent is obtained for the procedure. A single dose of prophylactic antibiotic is given. A commercially available, reusable cystostomy trocar made of stainless steel is used for the procedure. The instrument comprises two parts: an outer sheath and an inner obturator (Fig 1). The obturator has a built in small hole just proximal to the tip and another at the top, both connected through an internal channel. When the tip of the instrument enters the bladder, the flow of urine from the top hole confirms its position inside the bladder.

Once the obturator is removed, the crucial steps of the procedure are rapid catheter insertion by the assistant and immediate balloon inflation. As these two steps have to be performed very quickly, the procedure is practised first. Usually, a 14Fr Foley catheter is used for suprapubic drainage. An adequately distended bladder is confirmed by clinical examination and the catheter is checked for patency and balloon function before starting the procedure.

In the supine position, an incision (about 1cm) is made two fingerbreadths above the pubic symphysis after infiltrating the skin and underlying fascia with 10ml of 2% lignocaine solution. A 20G needle is advanced through the skin incision and aspirated to confirm the position of bladder. The incision is deepened up to the rectus sheath. The SPC trocar is advanced into the bladder with a gradual rotating motion of the hand, keeping a sustained pressure over it, the direction and depth being the same as determined by the needle, which is usually vertical or slightly towards the pelvis.

**Figure 2:**
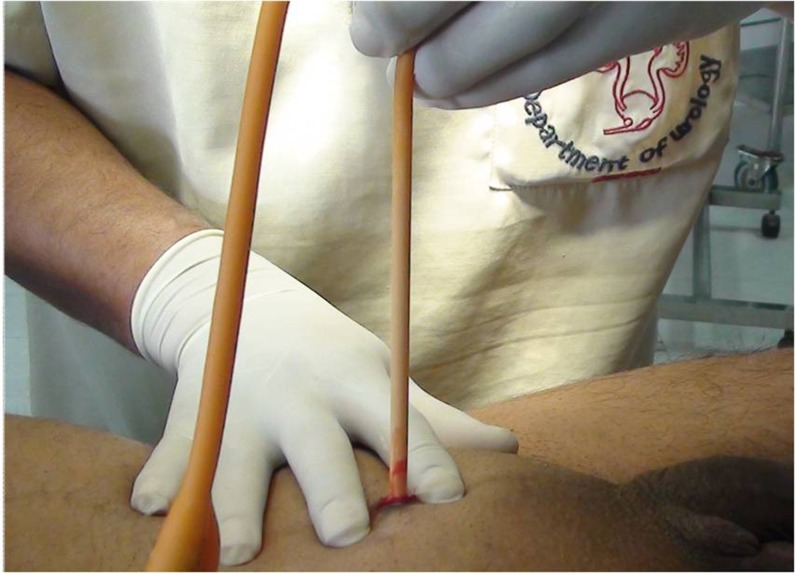
Gentle traction is applied on the catheter with countertraction on the abdominal wall by the opposite hand to tamponade the suprapubic catheter track.

Once the bladder is entered, the trocar is removed, holding behind the sheath inside the bladder. The assistant, who is ready with Foley catheter (with attached urine drainage bag and a prefilled syringe), inserts the catheter rapidly into the bladder through the sheath and inflates the balloon with 10ml of distilled water. As soon as the balloon is inflated, the sheath is removed and the catheter pulled back to tuck it against the abdominal wall (Fig 2). Gentle traction is applied on the catheter for about five minutes to ensure complete haemostasis. Simultaneously, the catheter is fixed to the anterior abdominal wall using a single silk 1/0 suture. A small sterile dressing is applied over it.

## Methods

Between May 2006 and December 2010, 72 men underwent percutaneous SPC using the above technique. The indications were acute urinary retention owing to traumatic urethral catheterisation (*n*=27) or urethral stricture (*n*=45). A perurethral catheter was either contraindicated or an attempt at passage had failed. All patients had a distended bladder at the time of the procedure, detectable on clinical examination. Ultrasonography guidance was not used in any case. All female patients, pelvic trauma patients and those having had previous lower abdominal surgery were excluded from the group of patients with the above technique and, instead, they underwent catheter insertion using other methods.

Over the same period, 17 men underwent SPC who did not fulfil the inclusion criteria. These comprised 11 patients suffering from pelvic trauma with urethral injury. Of these, one had associated bladder rupture, three had undergone previous lower abdominal surgery, two were obese patients in whom the bladder was not palpable and one had a small capacity bladder with continuous incontinence. None of these patients were subjected to blind SPC drainage because of the associated complicating factors. In 12 patients, SPC placement was performed under ultrasonography guidance, 4 patients underwent open SPC drainage and in the patient with the small capacity bladder the Seldinger technique was used.

## Results

The mean patient age was 42.4 years (range: 18–78 years). The procedure was performed successfully and no complications were encountered in any of the patients. There was no haematuria or catheter misplacement. The total duration of the procedure was less than five minutes (excluding the time of traction application, which is usually in place for five minutes to ensure complete haemostasis).

No complications were noted as all the included patients were straightforward with a clinically palpable bladder. In the patients with any of the complicating factors, the blind procedure was not followed and other methods were used safely, helping to avoid any complications.

## Discussion

SPC is a common procedure performed worldwide for bladder drainage when urethral access is not possible or advisable. It is an effective and even superior alternative to a chronic indwelling urethral catheter as it is easier to take care of and protects the urethra. Literature from different countries supports the role of SPC for continued bladder drainage. A study from Britain concluded that SPC is an effective and well tolerated method of urinary incontinence management in patients with neuropathic bladder dysfunction.^4^ These findings were in accordance with a study conducted in the US by Katsumi *et al*.^5^ In another study from Sweden, SPC proved to be superior to urethral catheterisation, even after transurethral resection of the prostate, at reducing the incidence of post-operative urethral stricture, improving the overall outcome of surgery.^6^ Similar results were supported in cases of acute urinary retention because of prostatic enlargement by Horgan *et al* from Ireland.^7^

Techniques for percutaneous SPC include direct puncture using a SPC trocar (disposable kits or reusable instruments),^8,9^ a modified trocar system using the Seldinger technique,^10^ cystoscopy guided SPC^3^ and SPC using fluoroscopy^11^ or ultrasonography guidance.^12^ The trocar SPC placement is a common method in clinical practice.

**Figure 3:**
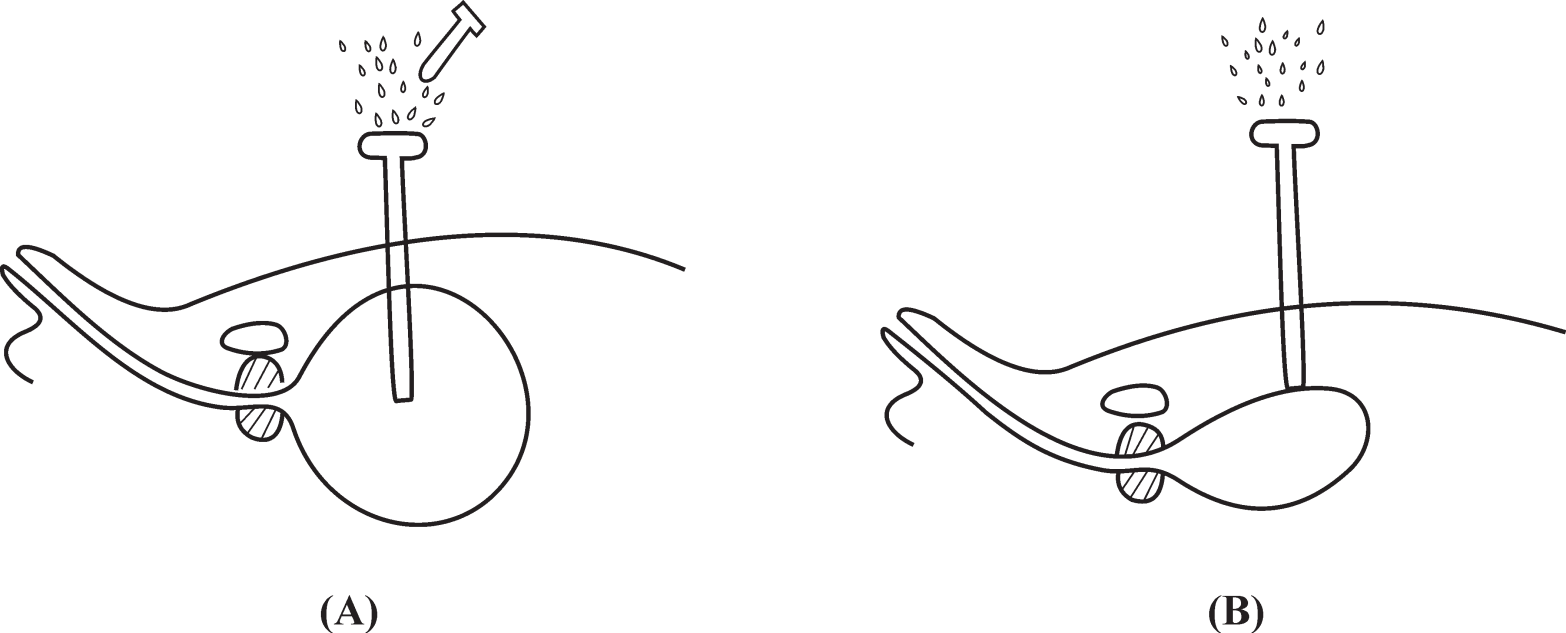
Mechanism underlying catheter misplacement: On obturator removal, urine leaks out rapidly from the sheath (A). This causes sudden bladder decompression, which may lead to sheath displacement out of the bladder wall (B), leading to catheter misplacement.

Two frequently encountered problems are track loss and haematuria. Ahluwalia *et al* reported SPC malposition/expulsion rate of 3% in a large retrospective series of 219 patients.^3^ However, it was a diverse group of patients and many had a neurogenic bladder. The reason for track loss is that once the obturator is removed, urine leaks out rapidly from the sheath, causing sudden bladder decompression, which may cause sheath displacement, in turn leading to catheter misplacement out of the bladder (Fig 3). This problem is more likely to occur with a less experienced surgeon as he or she may stop advancing the trocar immediately after entering the bladder for fear of injuring the posterior bladder wall or rectum. In such a situation, even slight bladder decompression may lead to track loss. To avoid this problem, we suggest advancing the sheath a little further inside the bladder while withdrawing the obturator.

Nevertheless, the key to avoiding this complication is the quick insertion of the Foley catheter once the obturator has been removed. This ensures that the catheter is inside the bladder with the balloon inflated before the bladder is collapsed.

As quick catheter insertion and immediate balloon inflation are the two vital steps of this procedure, they are explained clearly to the assistant before starting. Rapid balloon inflation excludes the possibility of catheter misplacement and ensures the correct position of the catheter inside the bladder. Once the balloon is inflated, it is tucked immediately against the abdominal wall, maintaining slight traction on the catheter to tamponade the suprapubic track. Haematuria after SPC is a commonly reported complication. Hasan *et al* reported haematuria in 25% of patients within the first 24 hours after the procedure.^13^ Although this haematuria is usually described as slight and insignificant, it can sometimes turn massive, necessitating clot evacuation.^14^ However, by placing traction on the SPC for five minutes, this complication is totally avoidable (Fig 2).

A minor procedure such as SPC is usually performed by resident surgeons and, if care is not taken in these steps, this minor procedure can be associated with significant complications. In spite of this, if a few important steps are performed carefully, even resident surgeons can perform SPC safely without complications. The most serious complications of percutaneous SPC include perforation of the peritoneum or intraperitoneal contents.^2^ Injury of adjacent organs is much more frequent when there is a history of previous lower abdominal surgery or when the bladder is not distended. In such cases, blind drainage procedures should be avoided.

In 1990 McMullin described his technique of percutaneous SPC using serial dilators passed over a guidewire,^15^ and the procedure is reported to be safe and effective. Kits using the Seldinger technique are available commercially. Use of a guidewire during catheter insertion has a much lower risk of track loss or catheter misplacement but cost and time becomes a major limiting factor for routine emergency practice and so this may only be used where cost and availability permits. With the reusable instruments in our technique, the cost of the procedure is reduced to a minimum. In the technique described by Papanicolaou *et al*, SPC is performed under fluoroscopy guidance after opacifying the bladder.^11^ Although it is shown to be an effective single-stage procedure, even for large bore cystostomy drainage, it is limited by unnecessary radiation exposure. High cost and time duration are other limiting factors.

Although ultrasonography guided SPC has been shown to be safe, effective and free of complications,^12^ there is no published evidence on its safety and efficacy in this role. Furthermore, routine ultrasonography guidance is not required in every patient and trocar SPC can be easily performed in well-selected patients. Patients with previous lower abdominal surgery or those with urinary retention because of pelvic trauma should undergo SPC by experienced surgeons under radiography guidance or even using an open procedure if required, to avoid the disastrous complications of adjacent organ injury, as open insertion is shown to be the safest option in these patients.

There are few conditions where SPC is contraindicated. Carcinoma of urinary bladder and uncorrected coagulopathy are generally accepted contraindications. In addition, SPC should be avoided in cases of sepsis of the anterior abdominal wall and in cases where a subcutaneous implant (eg a vascular graft) is present in the suprapubic area.

## Conclusions

Trocar SPC is a safe and effective bedside procedure for bladder drainage and can be performed by resident surgeons under training in selected patients. Few complications are associated with the procedure but these can be avoided easily with some careful steps as explained in our technique.
